# Differential diagnosis of white matter diseases in the tropics: An overview

**DOI:** 10.4103/0972-2327.48846

**Published:** 2009

**Authors:** Lekha Pandit

**Affiliations:** Department of Neurology, KS Hegde Medical Academy, Mangalore-575018, Karnataka, India

**Keywords:** Demyelinating disorders, white matter diseases

## Abstract

In hospitals in the tropics, the availability of magnetic resonance imaging (MRI) facilities in urban areas and especially in teaching institutions have resulted in white matter diseases being frequently reported in a variety of clinical settings. Unlike the west where multiple sclerosis (MS) is the commonest white matter disease encountered, in the tropics, there are myriad causes for the same. Infectious and post infectious disorders probably account for the vast majority of these diseases. Human immunodeficiency virus (HIV) infection tops the list of infective conditions. Central nervous system (CNS) tuberculosis occasionally presents with patchy parenchymal lesions unaccompanied by meningeal involvement. Human T cell leukemia virus (HTLV) infection and cystic inflammatory lesions such as neurocysticercosis are important causes to be considered in the differential diagnosis. Diagnosing post infectious demyelinating disorders is equally challenging since more than a third of cases seen in the tropics do not present with history of past infection or vaccinations. Metabolic and deficiency disorders such as Wernicke's encephalopathy, osmotic demyelinating syndrome associated with extra pontine lesions and Vitamin B12 deficiency states can occassionaly cause confusion in diagnosis. This review considers a few important disorders which manifest with white matter changes on MRI and create diagnostic difficulties in a population in the tropics.

## Introduction

Patients in developing countries consult doctors depending on the severity of their disease, proximity of specialist care and availability of affordable medical care. Medical records of past events are often unavailable and long term follow up data is poor. Laboratory evaluation has to be carefully planned based on history, circumstantial evidence and clinical examination. Very often the diagnostic workup of a patient is determined by the abnormalities seen on the MRI. Preceding history of fever, weight loss, and arthralgia, seizures at the onset of illness, palpable lymph nodes on examination and a raised erythrocyte sedimentation rate are some of the features that guide further investigations.

In this review, acute or subacute disorders presenting as white matter diseases clinically or those where imaging demonstrates predominantly white matter disorder have been included. Signs attributable to white matter involvement include involvement of corticospinal tracts, optic nerve, medial longitudinal fasciculus intracranially and partial cord syndromes affecting the spinal cord. Rarely these disorders may present with grey matter involvement in the form of seizures and cognitive impairment. The list of conditions causing white matter abnormalities on imaging is exhaustive and only those conditions which are common due to prevailing conditions in the tropics will be discussed [Table 1].

**Table 1:** Differential diagnosis of white matter diseases of the central nervous system

**Inflammatory demyelinating diseases**
Infectious - Human Immunodeficiency Virus and associated infections - Human T cell leukemia Virus - Tuberculosis - Syphilis - Viral encephalitis - Cystic inflammatory diseases - Neurocysticercosis - SSPE and other slow viral diseases - Lyme disease - Whipple's disease Inflammatory A Acute disseminated encephalomyelitis post infectious post vaccinial B Site specific demyelinating disorders-Transverse myelitis Neuromyelitis optica Brainstem encephalitis C Multiple sclerosis D Hashimoto's encephalopathy C Behcet syndrome E Neurosarcoidosis Metabolic/Nutritional - Wernicke's encephalopathy - Osmotic demyelination syndrome - Subacute combined degeneration (Vitamin B12 deficiency) Mitochondrial disorders. - Mitochondrial encephalopathy with stroke like episodes (MELAS) - Leigh's syndrome - Leber's optic atrophy Vascular Vasculitis - systemic lupus erythematosis, antiphospholipid antibody syndrome, Isolated angitis of the CNS Vasculopathy - Moya Moya disease, Retino coclear vasculopathy of Susac, CADASIL Cerebral venous thrombosis Neoplastic -Gliomatosis cerebri - Primary CNS Lymphoma - Paraneoplastic syndrome. Other Degenerative disorders - Spinocerebellar Ataxia Leukodystrophies Radiation Toxic- Carbon monoxide Clioquinol, Chemotherapy (Methotrexate,5 fluouracil) Reversible posterior leuco encephalopathy

## Infectious causes of white matter diseases

### Human Immunodeficiency Virus (HIV)

**Figure 1:**
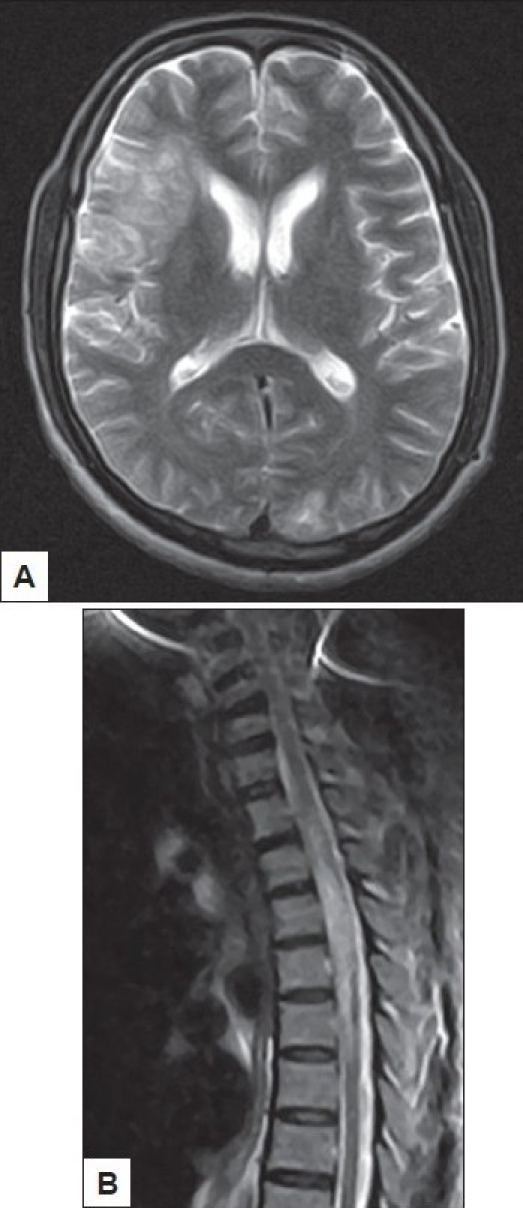
26 year male presented with acute onset paraparesis, retention of urine, and disorientation. One month earlier he had right focal motor seizures with transient post ictal weakness for which he had been investigated elsewhere with no details. MRI brain [Figure 1A] on T2W images showed large hyperintense lesions in the temporal and parietal subcortex on the right side with scattered small hyperintense lesions predominantly in subcortical white matter of the opposite hemisphere. MRI of the spinal cord [Figure 1B] on T2W images showed liner hyperintense lesion in the dorsal cord which enhanced moderately following intravenous contrast. He was HIV positive and succumbed shortly thereafter.Autopsy of the brain lesion confirmed the clinical diagnosis of progressive multifocal leucoencephalopathy

Patients may present for the first time with neurological deficits which necessitate imaging and then be found in the course of further work up to be HIV positive [Figure 1A and B]. HIV related CNS disorders are varied and may be due to the direct effect of the virus or a consequence of opportunistic infections, neoplasms and vascular diseases. Clinical findings are often nonspecific and subtle ranging from focal abnormalities and mild cognitive impairment to gross deficits and altered sensorium. Radiological features overlap the various disease subtypes. The abnormalities may be either diffuse or patchy.

## Diffuse white matter disease

### HIV Encephalopathy (HIVE)

Direct involvement of the brain by HIV is the most common cause of neurological disease.[1] Approximately 30% of HIV positive individuals manifest HIVE which is characterized by progressive dementia followed later by pyramidal and cerebellar dysfunction. Computerized tomography (CT) may show merely cerebral atrophy. Progression of disease results in the classical appearance of confluent, bilateral and symmetrical white matter lesions seen as diffuse white matter changes in the periventricular region and centrum semiovale, with relative sparing of the subcortical white matter and posterior fossa structures. On MRI these lesions have high T2 and isointense T1 intensity signals and there is no mass effect or contrast enhancement.

### Diffuse Cytomegalovirus (CMV) encephalitis

Both clinical and radiological features of CMV encephalitis mimic HIVE. It is very often detected as an autopsy finding.[2] Patients with CMV encephalitis have had acquired immunodeficiency syndrome (AIDS) for a longer duration and may have a more rapidly progressive dementia, lower CD4 counts and shorter survival period than HIVE patients.[3]

## Patchy white matter disease

### Progressive multifocal leucoencephalopathy

Progressive multifocal leucoencephalopathy (PML) is a rapidly progressive AIDS-defining disease of the CNS caused by the JC papovavirus.[4] There are no pathognomonic clinical features that define this condition. However focal white matter lesions in an immunocompromised patient should always raise the possibility of PML. The onset is insidious with progressive cognitive impairment combined with symptoms of white matter involvement such as visual deficits, language deficits, weakness and ataxia. While posterior fossa and spinal cord structures may be involved, the cerebral hemispheres bear the brunt of damage. Typical imaging findings are of patchy areas of low T1 signal and high T2 signal in the subcortical white matter. Lesions are often bilateral and asymmetrical although despite the name of the condition it may be unifocal. The lesions exert no mass effect. There is a parietal predominance; less common sites include the posterior fossa, basal ganglia and thalamus. Involvement of these central structures is due to contiguous spread along the white matter fibres that traverse them. Differentiation between HIVE and PML is important since highly active anti-retroviral therapy (HAART) therapy has been shown significantly to improve survival in patients with PML.[5]

## Herpes viral encephalitis

Clinically there are no pathognomic findings which distinguish Herpes simplex virus (HSV) encephalitis from other forms of central nervous system (CNS) infections. It produces a necrotizing encephalitis in the immunocompromised host. It is also more diffuse than the classic type, which has a predilection for the medial temporal and inferior frontal lobes. Poorly defined areas of edema with scattered foci of haemorrhagic signal are seen on imaging. In immunocompetent individuals who present with an encephalitic illness, treatment can be initiated with Acyclovir on the basis of MRI finding or lateralized deficits, provided cerebrospinal fluid (CSF) examination has reasonably excluded bacterial and tuberculous meningitis.

Varicella-zoster virus (VZV) also causes a multifocal encephalitis that predominantly affects the white matter. An enhancing margin may be seen at the edge of the lesion.

## Other infections

### CNS Tuberculosis

**Figure 2:**
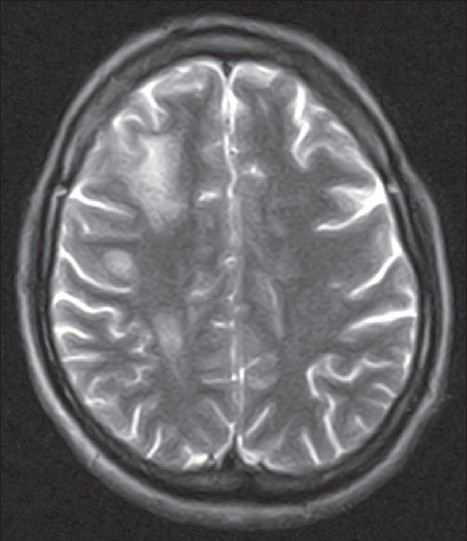
A 52 year old woman was investigated for headache and recent onset seizures. Her MRI of the brain showed three discrete hyperintense lesions in the right frontal, temporal and parietal subcortex which enhanced on contrast. Biopsy of the larger lesion revealed dense lymphocytic infilteration with scattered foci of plasma cells, epitheloid cells and Langerhan's giant cells and surrounding gliosis. A repeat MRI done 3 months after starting anti tuberculous therapy showed resolution of lesions

Tuberculous meningoencephalitis is an important differential diagnosis in countries with high prevalence for tuberculosis.[6] Diagnosis of CNS tuberculosis, especially tuberculous encephalomyelitis, is extremely difficult.[7] Clinical manifestations may vary from mild headache and low grade fever, to progressive alteration in sensorium, seizures and focal deficits. Fifty percent of cases do not have accompanying pulmonary tuberculosis. Definitive MRI signs of meningeal enhancement and hydrocephalus easily help to direct attention to a possible infectious etiology. However, focal parenchymal enhancements when seen alone on MRI [Figure 2], may be difficult to interpret.[8,9] Pathologically these lesions may show granulomatous vasculitis and spongiosis with reactive gliosis.[10,11] In such situations a mistaken diagnosis of ADEM or CNS vasculitis could lead to therapy with steroids which would cause a disastrous worsening of underlying disease. Disappearance of brain lesions after initiating anti tuberculous therapy is the final proof of diagnosis.[10]

### Neuro syphilis

In the post penicillin era, neurosyphilis presents more often with subtle neurological deficits or may be asymptomatic.[12] The duration of illness before developing neurological complications may be shortened and overlap syndromes are common especially in the background of HIV infection.[13] Clinical presentation may occasionally mimic acute transverse myelitis. Syphilitic meningomyelitis is still an important differential diagnosis for progressive myelopathy in the tropics.[14] Definitive diagnosis is made on CSF examination. Moderate lymphocytic pleocytosis, raised proteins and a positive VDRL test confirm the diagnosis. MRI of the spinal cord, especially in the dorsal segments shows hyperintense discrete lesions in T2W images which enhance with gadolinium.[15] On brain MRI multifocal white matter lesions may be seen[16] and occasionally positive oligoclonal bands are detected in the CSF[17] mimicking MS. Reversal of MRI abnormalities may occur following treatment.

### Human T cell leukemia virus (HTLV) infection

Exclusively seen in the tropics[18] it is an important differential diagnosis to be considered in patients presenting with progressive myelopathy.The latter is often referred to as tropical spastic paraplegia (TSP) or HTLV associated myelopathy (HAM).[19] HTLV1 seropositivity is high in the Caribbean islands and equatorial Africa and has been reported from parts of south America, Japan[19] and southern India.[20] Unlike HIV infection, neurological complications are low in seropositive individuals, myelopathy occurring in only 0.25% of cases.[21] The average age of onset is 40 years but can range from 20-70 years. The typical presentation is of progressive thoracic myelopathy associated with sensory and bladder dysfunction. Optic neuritis has been occasionally described. The clinical picture could be easily mistaken for primary progressive MS. CSF oligoclonal bands may be positive. MRI of the cord usually shows thoracic cord atrophy, but MRI brain may show white matter lesions similar to MS.[22,23]

### Cystic inflammatory lesions

Multiple cystic lesions in the brain may give cause for some diagnostic confusion. Occasionally post infectious demyelinating disorders may present as multiple cystic lesions. These lesions may have a complete ring enhancement pattern on contrast MRI.[24] Differential diagnosis for complete ring enhancing cystic lesions commonly include brain abscess, cysticercosis, tuberculoma and toxoplasmosis in a tropical country set up. Alternatively ADEM may present with cystic lesions that have an open ring enhancement pattern on MRI with gadolinium which may be of value in differentiating demyelinating from nondemyelinating diseases.[25,26]

Other infections like Lyme's disease are rare in the tropics and all cases of Whipple's disease reported so far have been from the west.

## Inflammatory demyelinating diseases

Many disorders are characterized by wide spread demyelination and include autoimmune, infectious, toxic, metabolic and vascular etiologies. Conventionally however, inflammatory demyelinating disorders specifically refers to multiple sclerosis (MS) and acute disseminated encephalomyelitis (ADEM).

### Acute disseminated encephalomyelitis

ADEM is a commonly made diagnosis in tropical countries where infections abound and vaccinations especially anti rabies vaccination (Semple's vaccine) are still freely in use. Traditionally ADEM has been described as a monophasic inflammatory central nervous system (CNS) disorder following viral infections and vaccinations.[27] In practice it is a loosely defined syndrome encompassing a variety of presentations all of which are of presumed post infectious etiology. These include monophasic pattern of disease with clinical and MRI evidence dissemination of lesions, site specific demyelinating disorders such as optic neuritis, acute transverse myelitis and brainstem encephalitis all of which involve a single clinical site but occasionally have MRI evidence of dissemination and lastly recurrent forms of ADEM. ADEM occurs in 30-40% of patients without preceding infections or vaccinations.[28,29] In the tropics, ADEM may occur following non viral infections too. The post malarial cerebellar syndrome which is a complication of severe Falciparum malaria is a typical example which resembles classical ADEM both clinically and on MRI.[30,31]

Usually after a self limiting illness, within a few days to weeks, ADEM develops as an acute multifocal neurologic illness. Signs and symptoms localizing to the brainstem, cerebellum, cerebral hemispheres, spinal cord and cranial nerves may occur alone or in varying combinations. There are no age specific differences in clinical presentations, though fever, headache and meningism are relatively infrequent in adults.[32]

ADEM shares remarkable similarities with multiple sclerosis in the sites of clinical involvement and MRI characteristics. MRI lesions in ADEM may be larger, more symmetric in the cerebral and cerebellar hemispheres and also involve the thalamus more often than in MS. Uniform enhancement of all lesions is considered to favour a diagnosis of ADEM since theoretically all lesions are of the same age. However the lesions in ADEM may evolve over days to weeks and in such situations, all lesions may not enhance.[33,34] Presence of Dawson's fingers (corpus callosum long axis perpendicular lesions) and periventricular lesions favour MS more than ADEM. In spite of these distinctions drawn on MRI between ADEM and MS, there is no one radiological feature that can confidently distinguish ADEM from the first attack of MS.[35,36]

ADEM as an entity has no definite diagnostic criteria and has a heterogenous clinical presentation. Most importantly it is a diagnosis of exclusion. Infections especially HIV, *Mycobacterium tuberculosis* and cystic infective brain lesions such as neurocysticercosis can mimic the disseminated form of disease. Investigations have to proceed beyond imaging and include serological tests for infections and often a lumbar puncture, before starting treatment.

### Acute Transverse myelitis (ATM)

Acute transverse myelitis is a heterogenous condition which typically presents with acutely or subacutely evolving spinal cord dysfunction. The dorsal spinal cord is most commonly involved with rapidly worsening paraplegia, bowel and bladder dysfunction and a definite sensory level. This clinical picture can be shared by variety of etiologies including vasculitis, neurosyphilis, tuberculous arachnoiditis and HIV infection, in a tropical setting. In addition, spinal form of MS has to be distinguished from post infectious ATM. Length of the spinal cord lesion as seen on the MRI is an important discriminator.[37] Clinical evidence of complete transverse myelitis coupled with MRI evidence of inflammation of the cord extending three or more vertebral segments is strongly against the diagnosis of MS.[38] Patients with acute partial transverse myelitis characterized by MRI lesions that are asymmetrically placed and spanning fewer than two vertebral segments in length may develop MS.[39]

### Neuromyelitis optica

**Figure 3:**
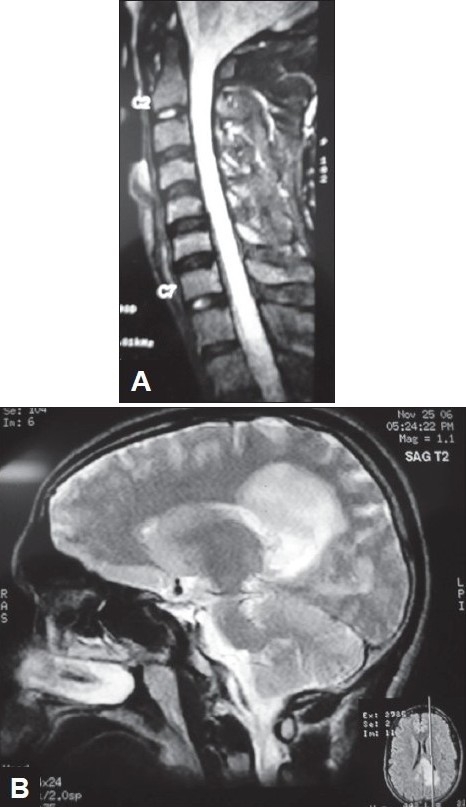
(A and B) 31 year old male presented with three episodes of recurrent myelitis interspersed with one episode of optic neuritis over a period of 18 months. Initial MRI brain was normal. MRI of the cervical cord showed (Fig. 3A) on T2 W image, a linear contiguous lesion spanning more than 3 vertebral segments suggestive of longitudinally extensive transverse myelitis. On his fourth hospital admission, he presented with headache, disorientation and worsening of lower limb power. MRI brain (Fig. 3B) showed large lumpy posteriorly situated subcortical lesions.

Conventionally neuromyelitis optica (NM0) is referred to a monophasic illness characterized by simultaneous occurrence of bilateral optic neuritis and transverse myelitis.[40] With the recent discovery of an immunoglobulin specific for neuromyelitis optica (NMO IgG) it has become a distinct entity.[41] New diagnostic criteria were established with the discovery of NMO IgG.[42] The clinical evidence of optic neuritis and myelitis should be supported by MRI of the spinal cord showing involvement of three or more contiguous segments. Longitudinally extensive transverse myelitis is a feature shared with post infectious transverse myelitis [Figure 3A]. It is therefore not surprising that NMO IgG has been found to be useful in predicting relapses in patients presenting with transverse myelitis.[43]

The role of MRI of the brain with intravenous Gadolinium in patients presenting with transverse myelitis cannot be over emphasized. Scott and colleagues[44] found that patients with acute partial transverse myelitis in the setting of normal brain MRI had a low rate of conversion (10%) to clinically definite MS after an average follow-up of 61 months. Brain MRI at onset is also an important criterion for the diagnosis of NMO. Brain MRI should be normal or have nonspecific lesions atypical for MS.[45] Brain lesions may accrue over a period of time and are typically seen in hypothalamus, thalamus and periependymal regions where aquaporin 4 expression is high[46] [Figure 3B].

### Brainstem encephalitis

**Figure 4:**
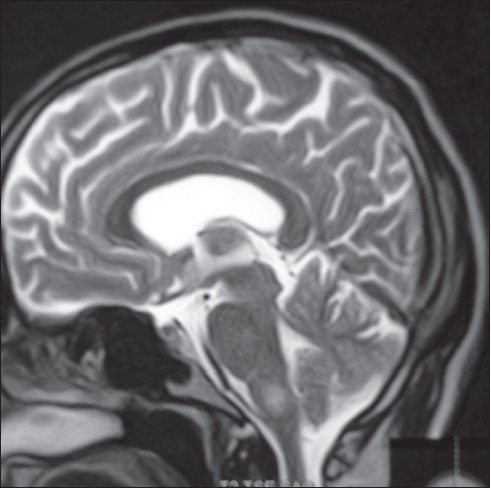
One week after a febrile illness, a 55 year old man developed neurogenic dysphagia accompanied by ataxia and drowsiness. Clinical examination revealed ophthalmoplegia, cerebellar signs and sluggish deep tendon reflexes. MRI of the brain showed a single hyperintense lesion in the pons, On T2 W images which moderately enhanced with contrast. He rapidly recovered following intravenous steroids

It is a post infectious syndrome characterized by progressive ophthalmoplegia, ataxia and altered sensorium. The MRI [Figure 4] shows evidence of focal brainstem demyelination in nearly 30% of patients.[47] Just as classical ADEM overlaps with peripheral neuropathy, brainstem encephalitis has been described with Guillain-Barré syndrome.[48] Nerve conduction studies may come in handy in differentiating first attack of MS from ADEM in this situation.

### Mutliple sclerosis

MS is considered to be uncommon in people of non caucasian origin.[49] Yet the prevalence of MS has dramatically increased in Asian countries such as Japan[50] and Iran[51] as evidenced by recent epidemiological studies. Hospital in patient statistics in India have shown a nearly fourfold increase in admitted cases over the last decade in comparison to the past.[52] The actual figures may be still under-represented in tropical countries for a variety of reasons. Awareness of MS among doctors and lay persons is still very low and specialist care is available to a limited few.[53] Prevalence studies of MS and long term follow up of patients presenting with clinically isolated syndromes suggestive of demyelination are lacking. Most studies point to a high involvement of optic nerve and spinal cord in MS from Asia as compared to the west.[54,55] In a tropical setting, MS has to be carefully differentiated from the pseudo relapses that occur with abrupt tapering of steroids in patients with ADEM[56] and from recurrent NMO, since the latter conditions are more likely to be prevalent than the former.

## Nutritional and Metabolic Disorders

### Vitamin B12 deficiency

**Figure 5:**
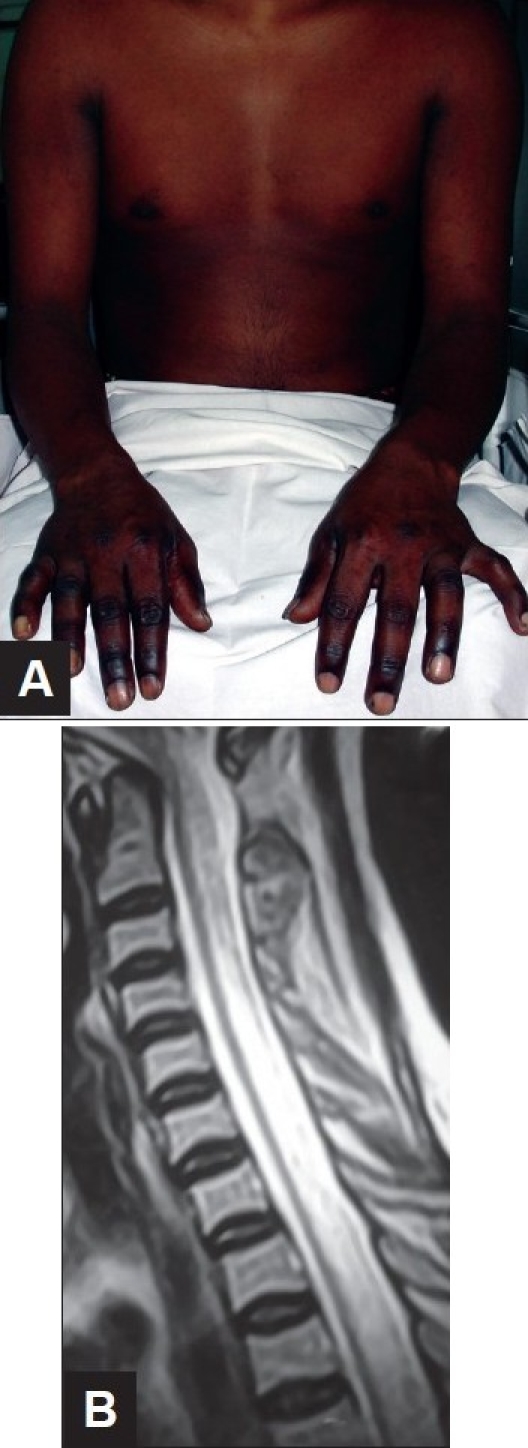
(A and B) 18 year old male was admitted with progressive parapalegia of 3 months duration. Earlier in the year he had similar weakness which improved partially when he was admitted to a local hospital for fever and upper respiratory infection (review of old prescriptions revealed that he had received vitamin supplements). Clinically he was anemic and had hyper pigmentation of distal extremities (Fig. 5A). Peripheral smear and bone marrow confirmed the diagnosis of megaloblastic anemia. MRI of the spinal cord (Fig. 5B) showed on T2W images, linear hyperintense lesion in the cervical and upper dorsal cord sparing the anterior column. He improved moderately with Vitamin B12 therapy

Vitamin B12 deficiency presents with progressive myelopathy which may sometimes appear to remit and relapse. Occasionally it presents as myeloneuropathy with cognitive dysfunction. In developing countries alcoholism, malnutrition and ileocecal tuberculosis are the common causes of Vitamin B12 deficiency. In a review of 63 case of sub acute combined degeneration seen in a period of 3 years, Aron *et al*,[57] reported that megaloblastic anemia was seen in all their patients. Striking skin changes especially hyper pigmentation of extremities and glossitis may be noted in nearly a third of patients [Figure 5A]. MRI demonstrates diffuse and multiple segment involvement of cervical cord in majority of the patients, although the pathology begins in the thoracic cord [Figure 5B]. Characteristic hyperintense signals on T2 W images associated with cord edema are seen predominantly involving the posterior column of the spinal cord.[58] Early recognition of this condition is essential, as it is reversible.

### Wernicke's encephalopathy (WE)

Traditionally described in the back ground of chronic alcoholism, Wernicke's encephalopathy may occur in a variety of other clinical settings.[59] The classical triad of ocular abnormalities, ataxia and altered sensorium may present in only a third of affected patients. MRI may show a range of abnormalities some of which overlap with ADEM.[60] These include bilateral and symmetric abnormal signal intensity on the T2-weighted and FLAIR images, especially in periventricular, periaqueductal regions and thalami. Signal changes may be noted in frontal cortex and deep white matter. Contrast enhancement may be seen in these regions and sometimes exclusively in the mammillary bodies.[61] Wernicke's encephalopathy is a medical emergency with a mortality rate ranging between 10% and 20% when untreated. When there is a clinical suspicion, trial of intravenous thiamine is essential. The, often dramatic, clinical improvement confirms the diagnosis.

### Osmotic demyelination syndrome (ODMS)

Seen in the background of chronic alcoholism and accompanying malnutrition, ODMS occurs in the setting of rapid changes in serum osmolality, typically during rapid iatrogenic correction of hyponatremia, less commonly in presence of other metabolic disturbances associated with osmotic shifts (hyperazotemia, hypokalemia, hyperglycemia, and/or ketoacidosis). The rapid changes in serum osmolality cause disruption of the brain-blood barrier with accumulation of hypertonic fluid in the extra-cellular space. Clinical symptoms, such as seizures, dysphagia, pseudo bulbar palsy, dysarthria, and movement disorders arise acutely after the occurrence of osmolality shifts. In the first week of illness, the MRI may be normal. By second week hyperintense signals may be seen on T2 weighted and FLAIR images involving central parts of pons. Contrast enhancement is generally not seen.[62] Extrapontine myelinolysis with or without pontine myelinolysis can occur. The brunt of the damage is in the basal ganglia and patients may present with altered sensorium, catatonia and extra pyramidal dysfunction. MRI scan may show hyperintense signals in the basal ganglia a feature shared with ADEM and arboviral encephalitis such as Japanese encephalitis.

## Mitochondrial disorders

Mitochondrial disease can present acutely and in the background of infection. It can sometimes cause a relapsing-remitting multifocal neurological picture and produce multifocal sub cortical white matter and basal ganglia lesions.[63]

**Figure 6:**
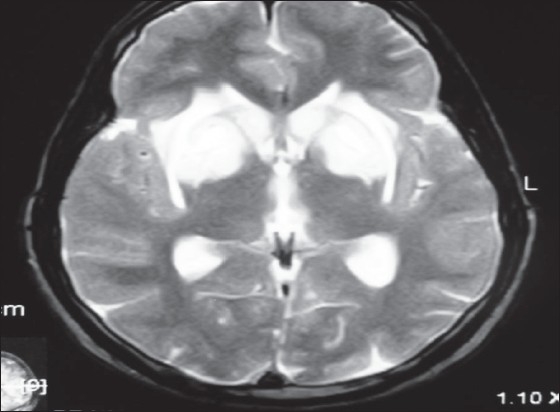
A 24 year old male presented with acute onset headache with drowsiness in the background of recent fever. On examination he had pupil sparing ptosis, vertical gaze paresis, mild sensory neural deafness and bilateral subtle pyramidal signs. MRI brain showed symmetric hyperintense lesions in the caudate nuclei and putamina extending into the thalami and periaqueductal regions, on T2W images (Figure 6). His serum and CSF lactic acid levels were raised.A quadriceps mucle biopsy revealed subsarcoplasmic accumuilation of mitochondria) associated with cytochrome oxidase negative fibres. No mitochondrial mutations were detected. Following intravenous Thiamine therapy he recovered rapidly

Leigh's disease typically presents with occulomotor dysfunction, bulbar and pyramidal dysfunction and obtunded sensorium. MRI findings are very characteristic with symmetric hyperintense signals on T2 weighted images in basal ganglia and brainstem[64] [Figure 6]. Serum and cerebrospinal fluid lactate estimation should be included in the work of such patients. Magnetic resonance spectroscopy though useful is not a commonly available diagnostic modality in a developing tropical country scenario.

Mitochondrial encephalopathy with lactic acidosis and stroke-like episodes (MELAS) may present like MS. Clinical features may occur in late childhood or even into adulthood. Transient visual loss, ataxia, hemiparesis often accompanied by headache and seizures are the common features. MRI may show multifocal hyperintense lesions on T2 weighted images in the white matter and basal ganglia. Lactic acidosis is best detected during an acute relapse.

## Other causes

CNS malignancies may mimic white matter disease, in the MRI era. Both CNS lymphoma and MS are multifocal white matter lesions that can share the features of similar CSF findings[65] and show dramatic response to steroids.[66] Glioma especially of the brainstem may appear as discrete white matter lesions which initially respond to steroids. Other lesions mistaken as white matter disease includes CNS angiitis complicating Hodgkin's disease and rarely paraneoplastic leucocytoclastic vasculitis. Degenerative disorders such as spinocerebellar degeneration and leukodystrophies, vasculitis associated with collagen vascular disease and isolated angiitis of the CNS are some of the other differentials for white matter disease.

## Conclusion

Clinicians working in a developing tropical country environment have limitations imposed by financial constraints that restrict the number of investigations that can be done in a given patient. In addition, in India, the field of neuroradiology is in its nascent phase and the mushrooming of sophisticated diagnostic tools such as the MRI are not always supported by skilled interpretation.

Clinical correlation of lesions seen on MRI is paramount to making a successful diagnosis. Imaging of the nervous system provides strong clues and guidelines for further evaluation [Table 2]. For example, the appearance of symmetric hyperintense lesions in the basal ganglia / thalamus could very well be due to ADEM, Leigh's disease, deep cerebral venous thrombosis, extrapontine myelinolysis or Japanese Encephalitis. Investigations have to carefully planned in order to be diagnosis specific and cost effective. The setting in which the disease evolved and the presence of extra CNS symptoms such as arthritis, fever, hyponatremia, alcohol dependence are important in determining underlying etiology. The initial work-up should include serum testing for inflammatory screening with erythrocyte sedimentation rate, C-reactive protein (CRP), antinuclear antibody and anti-neutrophil cytoplasmic antibodies (ANCA); infectious screening for syphilis, HIV and tuberculosis; and metabolic screening for vitamin B_12_ deficiency and thyroid dysfunction. Interpretation of results have to be done cautiously. For example, oligoclonal bands in the CSF and elevations in IgG index are nonspecific markers of CNS inflammation that are described in a multitude of infectious, inflammatory, and neoplastic CNS diseases in addition to MS[67] and should not mislead investigations. Steroids are the empirical treatment given in most settings. Response to steroids does not necessarily confirm the diagnosis of demyelinating disease since several other conditions including CNS vasculitis, sarcoidosis and CNS lymphoma show reponse.

**Table 2:** Differential diagnosis based on magnetic resonance imaging

White matter alone	MS
	CADASIL
Grey and white matter	ADEM
	Ischemia /anoxia
	PRES
	Neoplasm
Symmetric lesions	ADEM
	HIVE
	PRES
	Leucodystrophy
	Leigh's syndrome
Asymmetric lesions	PML
Contrast enhancement -	Open ring enhancement -ADEM
Ring enhancement -	ADEM, Infective granuloma
No enhancement	HIVE,
	PML,
	Toxic,
	paraneoplastic,
Site specificity -	Medial temporal and inferior frontal -HSE
	Temporal pole and external capsule-
	CADASIL
	Bilateral Pareito- Occipital - PRES
	Basal ganglia and thalamus - ADEM
	Extrapontine myelinolysis
	Deep venous system infarcts
	Japanese encephalitis
	Leigh's syndrome
Periaqueductal midbrain -	WE, Leigh's disease.
	Mamillary bodies - WE
Pons -	Central pontine myelinolysis
	Mass effect, Infective granuloma
	Neoplasm
	Tumifactive demyelination

MS= Multiple sclerosis, CADASIL= Cerebral autosomal dominant arteriopathy with subcortical leukoencephalopathy, ADEM= Acute disseminated encephlomyelitis, PRES= Posterior reversible encephalopathy syndrome, HIVE= Human Immuodefficiency virus encephalitis, PML= progressive multifocal leukoencephalopathy.HSE= herpes simplex encephalitis, WE= Wernicke's encephalopathy
